# Gender-Based Differential Item Function for the Positive and Negative Semantic Dimensions of the Relationship Satisfaction Scale with Item Response Theory

**DOI:** 10.3390/bs13100825

**Published:** 2023-10-07

**Authors:** Sai-fu Fung, Jiahui Jin

**Affiliations:** 1 Department of Social and Behavioural Sciences, City University of Hong Kong, Hong Kong, China; 2 Wee Kim Wee School of Communication & Information, Nanyang Technological University, Singapore 639798, Singapore; carolejin7@gmail.com

**Keywords:** relationship satisfaction, MIRT, DIF, PN-SMD, Chinese, IRT

## Abstract

Relationship satisfaction is at the core of a robust social life and is essential to mental health. The positive and negative semantic dimensions of the relationship satisfaction (PN-SMD) scale is considered in the field of relationship studies to be a reliable tool for assessing the quality of a person’s interpersonal relationships. This study evaluated the psychometric properties of the PN-SMD scale by conducting multidimensional item response theory (MIRT) and differential item functioning (DIF) analyses, both of which are emerging assessment methods that focus on individual items. We recruited 511 Chinese undergraduate students for this study. Construct validity, internal consistency, and concurrent validity were assessed, and MIRT and DIF analyses were conducted. Five of the 14 items were found to have gender-based DIF traits, affecting the scale’s construct validity. A revised nine-item scale (DIF items excluded) had a significantly better model fit and demonstrated comparable concurrent validity to the original scale. The implications of our results and future research directions are discussed.

## 1. Introduction

Relationship satisfaction refers to a person’s own assessment of their interpersonal relationships and is regarded as an essential variable in relationship research [1]. Satisfaction in interpersonal relationships is an important component of mental well-being. Vanhalst, et al. [2] suggested that poor-quality relationships can lead to loneliness, which typically arises when one’s interpersonal relationships do not fulfil expectations [3]. The recent literature also suggested the quality of social relationships has been shown to be correlated with life satisfaction [4,5], positive affect [6,7], self-esteem [8,9], and self-efficacy [10]. As relationship satisfaction and relationship quality are very similar measures, a fact that is widely recognized in marriage-related research [11,12], we assume that satisfaction and quality are synonymous in the context of relationships [13]. However, no broadly accepted scale that measures relationship quality has been developed thus far.

In the early stages of the development of relationship quality scales, scholars tended to define the scales as unidimensional models (i.e., from dissatisfied to satisfied), in which the total score was thought to reflect an individual’s evaluation of his or her social relationships. Examples of these scales are the marital adjustment test (MAT) [14], the dyadic adjustment scale (DAS) [15], and the couples satisfaction index (CSI) [12]. Although these models are widely used and have been shown to demonstrate good psychometric properties, their unidimensional structure has been repeatedly challenged [16,17]. Unidimensional measures of relationship quality are now believed to obscure various characteristics in interpersonal relationships [18,19], as most relationships have both satisfactory and unsatisfactory aspects [20].

Based on the argument above, Fincham and Linfield [7] proposed a two-dimensional scale with six items assessing relationship quality. This measure, the positive and negative quality in marriage scale (PANQIMS), demonstrated adequate psychometric properties. PANQIMS allowed for the measurement of ambivalence and indifference, concepts that cannot be measured in single-dimensional models. Mattson, Rogge, Johnson, Davidson and Fincham [1] highlighted some of the limitations of PANQIMS (e.g., failure to account for conflicting attitudes and inconsistency in the target of assessment) and proposed a 14-item scale called the positive and negative semantic dimensions of relationship satisfaction (PN-SMD) based on the semantic differential technique [21]. The PN-SMD comprises two seven-item subscales that measure the positive and negative aspects of a relationship separately. Each subscale includes seven adjectives that are positive or negative according to the subscale. The instrument shows adequate incremental validity [1]. The PN-SMD has been used to examine deviant internet behavior among Chinese adolescents [22] and the mediation effect between problematic internet usage and self-esteem among Chinese undergraduates [23]. Although the PN-SMD showed good internal consistency in previous research (α = 0.92–0.93) [24], it showed deficiencies in individual item evaluation when item response theory was used [13]. In previous assessments using IRT, a unidimensional model was adopted, although PN-SMD is clearly a two-factor model. This may lead to concerns about applicability. It is therefore important to revisit the PN-SMD using multidimensional item response theory (MIRT) [25].

Item response theory (IRT), also known as latent trait theory, is designed to evaluate each item in a scale individually, using a statistical model [26]. There have been several applications of IRT, such as the CSI scale by Rogge, Fincham, Crasta and Maniaci [13] and the PN-RQ scale by Funk and Rogge [12], in the field of developing relationship scales. Funk and Rogge [12] suggested to analyzing the two subscales separately for the two-dimensional PN-SMD and PN-RQ. As such, MIRT is a more appropriate model, as it involves an IRT analysis of multiple scales [25] to assess the two-factor PN-SMD. 

Differential item functioning (DIF) within the IRT framework is becoming more popular for evaluating a scale’s validity in education, psychology, and clinical settings [27,28]. DIF assesses whether different subgroups respond differently to each item [29]. A more recent and popular approach is multiple indicators and multiple causes (MIMIC) modeling for the detection of DIF [30]. The MIMIC model is a specialized version of structural equation modeling (SEM) that incorporates causal variables, or covariates, into a confirmatory factor analysis model [31]. According to Cheng, Shao and Lathrop [30], DIF can be understood as a model mediated by groups. There has been a scarcity of DIF analysis conducted on scales measuring relationship quality in the existing literature. The invariance of PN-SMD has been tested by Zeng, Zhang, Fung, Li, Liu, Xiong, Jiang, Zhu, Chen and Luo [23], who examined the configural invariance, metric invariance, and scalar invariance of the whole scale. Still, an investigation of the invariance for each item is warranted because the level of information contained in the individual items in the PN-SMD was found to be inadequate [13], and the model fits reported in previous studies were also unsatisfactory [23], though the internal consistency was good (α = 0.92–0.93) [24]. When employing a MIMIC model, both the measurement model and the structural model can be used to evaluate the direct effect of a covariate that defines group membership (e.g., gender) on factor means and factor indicators (items) [31]. This method can help to identify any gender-based DIF traits in the PN-SMD.

Based on the above controversies regarding the PN-SMD, it is important to re-evaluate the psychometric properties of both the whole scale and its individual items. MIRT and DIF are seldom-used scale assessment tools that provide superior assessment of individual items. This work will generate new insights that cannot be obtained using the typical mean groups difference approach.

## 2. Methods

### 2.1. Participants

Our cross-sectional research was conducted between April and May 2019 as part of a larger study focused on examining the relationship between quality of life and internet usage at a university in Guangzhou, China. Five hundred and eleven undergraduate students (18–23 years old) were recruited (refer to Table 1) through the university’s intranet system, utilizing a smartphone-based self-report application. The sample represented the demographic profile of the university population. Prior to participating in the study, all participants provided informed consent.

The research procedures adhered to the relevant regulations outlined in the current versions of the Statistics Law of the People’s Republic of China and the Declaration of Helsinki.

### 2.2. Measures

The PN-SMD scale is a relationship satisfaction scale. Participants are required to rate 14 items, including 7 positive qualities (interesting, full, sturdy, enjoyable, good, friendly, and hopeful) and seven negative qualities (bad, lonely, discouraging, boring, empty, fragile, and miserable) on an 8-point Likert-type scale, ranging from 0 (not at all) to 7 (always) [1]. 

The positive and negative affect schedule (PANAS) [32] is a 20-item measure designed to self-evaluate positive affect (PA) and negative affect (NA) using a 5-point Likert-type scale. The overall reliability of PANAS is 0.846. In this study, the Cronbach’s α values for the PA and NA subscales (10-item each) were 0.802 and 0.884, respectively, which aligns with those reported in other PANAS studies conducted in the Chinese context [33,34,35].

The brief resilience scale (BRS) [36], is a self-reported measure to assess the perceived ability to recover from stress, comprising six 5-point Likert-type items ranging from 1 (does not describe me at all) to 5 (describes me very well), e.g., ‘I tend to bounce back quickly after hard times’. The scale demonstrated good internal consistency in this study (α = 0.708). The Chinese version of the BRS was translated and validated by Fung [37].

To assess the participants’ satisfaction with their lives, we utilized the satisfaction with life index (SWLS) [38]. SWLS items are rated on a 7-point Likert-type scale ranging from 1 (strongly disagree) to 7 (strongly agree). The total score, derived from summing all item scores, ranges from 5 to 35, with higher scores indicating greater life satisfaction. The Cronbach’s α in this study was 0.819, which is comparable to values reported by Diener, Emmons, Larsen and Griffin [38] and Kong, et al. [39] (α = 0.81–0.89).

The Chinese version of the Rosenberg self-esteem scale (RSE), validated by Wu, et al. [40], was employed to measure participants’ self-esteem. This scale consists of 10 items rated on a 4-point Likert-type scale ranging from 1 (strongly disagree) to 4 (strongly agree), for example, ‘I wish I could have more respect for myself’. Song, et al. [41] reported a Cronbach’s α of 0.83 for this scale among Chinese university students. In the present study, the Cronbach’s α was 0.755.

The general self-efficacy scale (GSE) [42], translated by Zhang and Schwarzer [43], comprises 10 items, each rated on a 4-point Likert-type scale ranging from 1 (absolutely incorrect) to 4 (absolutely correct), e.g., ‘I am confident that I could deal efficiently with unexpected events’. The scale demonstrated good internal consistency in this study, with a Cronbach’s α of 0.884, which is consistent with recent research conducted by Zeng, et al. [44].

### 2.3. Data Analysis

IRT is a testing model that is based on the relationship between respondents’ scores on an item and their level on the overall score on the scale [45,46]. According to Brennan [47], IRT focuses on item responses, whereas classical test theory (CTT) focuses on test or form scores. There are different assumptions and features related to the IRT and CTT, such as in the issues related to forms and parallelism, true score, and assumptions’ primary strengths. As CTT is beneficial in evaluating brief instruments [48,49], the MIRT model was adopted in this study to calibrate item parameter estimates and assess how participants responded to item-level stimuli [25]. MIRT combines IRT and factor analysis, enabling the analysis of multi-factor models. The MIRT model calculates an item discrimination index (a parameter) for each factor and intercept parameters (d parameters). The difficulty (threshold) values, which reflect the probability that a person will score above or below a given threshold, were computed using the following equation: a parameter * difficulty (threshold) values = intercept. The criterion (a parameter > 1.00) was used [50,51], a practice widely adopted in recent IRT analyses [52,53,54]. Values > 1.70 are considered very high [55]. 

DIF was calculated using the MIMIC method with scale purification, which controls false-positive rates and yields higher true-positive rates [56]. Recent simulation study also suggested that a stepwise purification procedure is suitable for the sample size and number of items of the scale in the current study [57]. Following Byrne [58], because the scale is based on categorical data, the weighted least squares (WLS) approach was used to estimate the parameters. This involved (a) testing each item for DIF one at a time, using all other items as the anchor; and (b) subsequently using a purified anchor to test all remaining items for DIF, repeating this process until the same set of items was detected as showing DIF for two successive iterations [30]. The following criteria indicated DIF items: −1.96 < z value < 1.96; *p* value < 0.05 [59]. 

To evaluate the construct validity and verify the shortened version [60], confirmatory factor analysis (CFA) with the DWLS estimator [61] was used to evaluate the construct validity of the PN-SMD scale. The following criteria indicated a good model fit: comparative fit index (CFI) > 0.95, Tucker–Lewis index (TLI) > 0.95, root mean square error of approximation (RMSEA) < 0.06, standardized root mean square residual (SRMR) < 0.06, and χ^2^/df ≤ 3 [62].

Concurrent validity was evaluated using other construct-related scales, consistent with the previous literature. Relationship satisfaction has been shown to be significantly correlated with positive or negative affect [6,7], resilience [63], satisfaction with life [4,5], self-esteem [8,9], and self-efficacy [10]. Hence, we used PANAS, BRS, SWLS, RSE, and GSE to assess the convergent and divergent validity of our proposed scale adaptation.

All of the above analyses were computed with SPSS version 26.0, in the R computing environment (4.1.1) [64] with the lavaan package 0.6–9 [65] and Mplus (8.6) [66].

## 3. Results

### 3.1. MIRT Results

Table 2 shows the PN-SMD item parameter estimates for MIRT. The items had parameters for their own dimension between 1.969 and 3.9. All of these exceeded 1.7, which is regarded as a high value for a slope parameter [55]. This means that all of the items discriminated well between low and high levels of positive and negative relationship satisfaction. 

Although the scale comprised 8-point items, the fact that no respondents selected option 0 resulted in only 6 intercepts being reported. For the graded response model, a minimum distance of 0.3 between adjacent intercepts is required to ensure that closer categories will not be selected often [25]. As shown in Table 2, the distance between intercepts was satisfactory.

Difficulty or ‘threshold’ values (b parameters) were calculated using the equation above. For example, the threshold parameters of PN-SMD 1 were −2.287, −1.464, −1.107, −0.003, 0.670, and 1.963. These parameters signify the cut-off points between item categories.

### 3.2. DIF Results

Before conducting the DIF analysis, we compared the mean PN-SMD scores using a single-df analysis of variance (ANOVA). No statistically significant differences were found in subscale scores between gender-based groups (*p* = 0.516/0.710 > 0.05). Table 3 shows the results of DIF items in the last iteration of the MIMIC model using the scale purification method. The items in Table 3 show DIF in two consecutive iterations, with PN-SMD 9 presenting marginal values. As the β values of PN-SMD 1, PN-SMD 2, PN-SMD 5, and PN-SMD 10 were negative, these items were regarded as in favor of the focal group (female). Conversely, PN-SMD 9 is in favor of the reference group (male). Women were more likely to use the adjectives ‘interesting’, ‘full’, ‘good’, and ‘discouraging’ when describing their relationships, whereas men were more inclined to say they felt lonely. For detail corresponding z values in each iteration, please refer to Appendix A.

### 3.3. Construct Validity

The 14-item PN-SMD failed to show an acceptable model fit in this sample of Chinese undergraduate students (see Table 4). After deleting the DIF items identified above, the new nine-item model demonstrated a significant improvement over the original model. However, this new model did not fulfil some of the cut-off criteria for a good model fit (χ^2^/df, RMSEA, and TLI). Stability is an important element of belonging [67], and there is a clear correlation between belonging and enjoyment [68]. To evaluate this possible correlation, we correlated PN-SMD 3 (stable) with PN-SMD 4 (enjoyable). The nine-item model (P-SMD: items 3, 4, 6, and 7; N-SMD: items 8, 11, 12, 13, and 14) correlated error terms showed satisfactory values, with the exception of χ^2^/df (with 3.43 > 3). According to Marsh and Hocevar [69], χ^2^/df < 5.00 still can be regarded as a parsimonious fit. Thus, the final model can be considered statistically acceptable. 

### 3.4. Concurrent Validity

As shown in Table 5, the new subscales (with deleted DIF items) showed satisfactory convergent and divergent validity according to Pearson’s correlation coefficient, with the new scale being comparable to the original scale (*r* = 0.951/0.98, *p* < 0.01).

## 4. Discussion

In the present study, we evaluated each item in the PN-SMD scale proposed by Mattson, Rogge, Johnson, Davidson and Fincham [1] using MIRT and DIF. With reference to the Web of Science database, this is the first time that each item on the scale has been assessed individually. 

Based on the results of our MIRT analysis, all of the items in both subscales showed a good ability to distinguish between different levels of positive or negative relationship satisfaction. The slope parameters of the positive subscale (M = 2.51; SD = 0.23; range 2.290–2.893) and the negative subscale (M = 3.06; SD = 0.69; range 1.969–3.900) reached the value of a parameter > 1.7 and are therefore considered highly discriminated [55]. These parameters are comparable to the factor loadings on each dimension. This finding is in line with the idea that a higher parameter value indicates a stronger link to the structure [70], as the ‘mirt’ package [25] provides intercepts (d parameters) rather than difficulty or ‘threshold’ values (b parameters). Threshold parameters (b parameters) were found to be unevenly distributed across the trait range. The items on the positive subscale (for example, PN-SMD1: “My relationship is interesting”) showed a positive skew, while items on the negative subscale (such as PN-SMD9: “My relationship is lonely”) exhibited a negative skew. This means that more respondents were likely to endorse positive adjectives when describing relationships [71], which is consistent with the findings of Rogge, Fincham, Crasta and Maniaci [13]. In summary, all 14 items in the PN-SMD showed sufficient discrimination and difficulty in the MIRT model.

The results of DIF analysis provide new insight into the function of PN-SMD items across different gender groups. Of the 14 PN-SMD items, five exhibited DIF in favor of female participants (PN-SMD 1: interesting, PNSMD 2: full, PN-SMD 5: good, and PN-SMD 10: discouraging), while one exhibited DIF in favor of male participants (PN-SMD 9: lonely). When discussing DIF items, it is important to note that items showing DIF are not necessarily biased [72]. Zieky [73] suggested that judgmental reviews are required when converting statistical differences into practical significance. As the attributes of the MIMIC model and the detected DIF are uniform, MIRT shows a significant group difference in the item intercept, or difficulty [74]. In this study, female participants had higher scores on most items marked as DIF. This is consistent with evidence that women have a better ability to identify and name their feelings than men [75,76]. Although this finding does not explain why these particular items showed differences in item function, it does support the possibility that there may be a female bias. Consistent with the higher scores of male participants for PN-SMD 9 (lonely), some studies have suggested that men report more loneliness. Women tend to prefer dyadic relationships, which may lead to a stronger connection with partners, whereas men tend to interact in groups of three or more people [77,78,79]. Borys and Perlman [80] found that men reported a higher level of loneliness than women, a finding replicated by Stokes and Levin [79]. In summary, the results of this study indicate that the PN-SMD exhibit gender-based differential item functioning (DIF) characteristics. It is important for future studies to take this into consideration when utilizing this scale.

Another major contribution of this study is to propose an adapted version of nine-item PN-SMD that omits the gender-biased DIF items. According to Teresi, et al. [81], it is sometimes appropriate to remove an item with DIF or to flag it as an item that should not be used for certain groups. We ran CFA on the original scale and found that without the DIF items, the model fit improved considerably (see Table 4). As the indices did not reach all of the cut-off values listed, we further correlated PN-SMD 3 (stable) and PN-SMD 4 (enjoyable) based on the modification index. The final model (removed items 1, 2, 5, 9, and 10) showed a satisfactory fit, demonstrating that deleting DIF items can be a fruitful measure. The results of a Pearson’s correlation analysis showed that the revised model had adequate concurrent validity and the coefficients were comparable to those of the initial model. 

This study has several limitations. The first is that the MIMIC model adopted here only examines uniform DIF [74], which means that non-uniform DIF may have existed but may not have been detected. In future research, scholars can adopt other methods of assessing DIF that are able to detect non-uniform DIF, such as logistic regression and the IRT model [74,82,83,84,85] and quantify the impact of DIF on mean comparisons by a variance measure by linking errors, either by analytical treatments [86] or with resampling techniques [87]. Second, the probability of Type I errors in this study was inflated because we did not test the clinical meaning of DIF using qualitative methods, which is considered a best practice [88]. This limitation could be improved in future studies by using mixed methods and the Bonferroni correction [89] as well as the simultaneous item bias test (SIBTEST) to account for multilevel data structures with small sample context [90,91]. Given the importance of interpersonal relationship satisfaction to a person’s mental health, it is of obvious practical significance to accurately measure the quality of interpersonal relationships in different groups. Removing gender-biased items makes this scale more suitable in various contexts.

## 5. Conclusions

The validity and rationality of relationship satisfaction, an important concept in psychological well-being, is still widely debated. MIRT and DIF are increasingly being used to assess psychometric properties, and this study represents the first time these methods have been applied to a scale related to relationship satisfaction. The main contribution of this study is an analysis of the psychometric properties of the PN-SMD from the perspective of individual items. Based on our findings, we propose a nine-item scale that omits the gender-biased DIF items. The new scale shows satisfactory construct validity that is comparable to the original scale. It is suggested that this nine-item scale could be adopted in subsequent studies related to relationship satisfaction, especially those working with a Chinese sample.

## Figures and Tables

**Table 1:** Participant Demographic Characteristics.

Variable	Respondents (n = 511)
Age mean (SD)	20.41 (2.49)
Gender n (%)	
Male	74 (14.5%)
Female	437 (85.5%)

**Table 2:** MIRT Estimates for Item Discrimination and Intercept Parameters.

Item	$a_{1}$	$a_{2}$	$d_{1}$	$d_{2}$	$d_{3}$	$d_{4}$	$d_{5}$	$d_{6}$
PN-SMD 1	2.442	–	5.586	3.575	2.703	0.076	−1.635	−4.794
PN-SMD 2	2.386	–	5.620	3.422	1.873	−0.360	−2.020	−4.803
PN-SMD 3	2.268	–	5.953	3.549	2.178	0.255	−1.608	−4.355
PN-SMD 4	2.893	–	7.610	5.520	3.638	0.916	−1.239	−4.900
PN-SMD 5	2.679	–	6.996	4.676	2.962	0.141	−1.833	−5.148
PN-SMD 6	2.290	–	6.736	4.765	3.285	1.278	−0.337	−3.583
PN-SMD 7	2.590	–	6.679	4.876	3.315	0.827	−1.018	−4.165
PN-SMD 8	–	1.969	2.490	0.105	−1.474	−2.963	−4.551	−6.364
PN-SMD 9	–	2.491	3.419	0.813	−0.746	−2.288	−3.797	−5.626
PN-SMD 10	–	3.900	3.979	−0.147	−2.129	−4.541	−6.351	−8.718
PN-SMD 11	–	3.350	3.864	0.052	−1.774	−3.594	−5.787	−8.458
PN-SMD 12	–	3.590	3.844	−0.246	−2.150	−4.300	−6.198	−8.425
PN-SMD 13	–	2.677	3.058	0.244	−1.232	−2.789	−4.562	−7.846
PN-SMD 14	–	3.433	3.260	−0.926	−2.553	−4.598	−6.432	−7.999

Note. $a_{1}$ = discrimination index for positive subscale, $a_{2}$ = discrimination index for negative subscale, $d_{n}$ = intercepts.

**Table 3:** DIF Items and the Corresponding z Values in the Last Iteration.

DIF Item	β	σ (β)	z	*p*
PN-SMD 1 (interesting)	−0.122	0.033	−3.644	0.000
PN-SMD 2 (full)	−0.080	0.032	−2.476	0.013
PN-SMD 5 (good)	−0.074	0.037	−1.973	0.048
PN-SMD 9 (lonely)	0.069	0.029	2.347	0.019
PN-SMD 10 (discouraging)	−0.050	0.024	−2.100	0.036

Note. β = severity parameter, σ (β) = standard error (severity parameter), z = β/σ (β).

**Table 4:** Confirmatory Factor Analysis of PN-SMD models.

Model	χ^2^	df	χ^2^/df	RMSEA [90% CI]	CFI	TLI	SRMR
14-item model	463.414	76	6.10	0.100 [0.091~0.109]	0.918	0.902	0.044
9-item model	126.580	26	4.87	0.087 [0.072~0.102]	0.960	0.945	0.031
9-item model *	85.733	25	3.43	0.069 [0.053~0.085]	0.976	0.966	0.024

Note. Nine-item model *: correlated PN-SMD 3 and PN-SMD 4.

**Table 5:** Correlation for seven-item subscales in relation to newly subscales.

Scale	P-SMD (4 Item)	N-SMD (5 Items)	P-SMD (7 Items)	N-SMD (7 Items)
	α = 0.857	α = 0.889	α = 0.904	α = 0.921
P-SMD (4 item)	–			
N-SMD (5 item)	−0.457 **	–		
P-SMD (7 item)	0.951 **	−0.486 **	–	
N-SMD (7 item)	−0.464 **	0.981 **	−0.502 **	–
Positive Affect	0.307 **	−0.125 **	0.377 **	−0.131 **
Negative Affect	−0.238 **	0.447 **	−0.241 **	0.451 **
Resilience	0.389 **	−0.392 **	0.393 **	−0.388 **
SWLS	0.390 **	−0.263 **	0.438 **	−0.276 **
RSE	0.376 **	−0.427 **	0.420 **	−0.443 **
GSE	0.237 **	−0.184 **	0.280 **	−0.172 **

** Correlation is significant at the 0.01 level (2-tailed).

## Data Availability

Correspondence and requests for materials should be addressed to S.F.

## Appendix A. The Corresponding z Values in Each Iteration

**z-Value**								
**Identified Anchors**	**PN-SMD 2**	**PN-SMD 4**	**PN-SMD 5**	**PN-SMD 7**	**PN-SMD 11**	**PN-SMD 12**	**PN-SMD 13**	**PN-SMD 14**
**PN-SMD 1 (interesting)**	−2.454 *	−4.534 ***	− 2.891 **	−4.345 ***	−4.107 ***	−4.140 ***	**−3.692 *****	**−3.644 *****
**PN-SMD 2 (full)**	/	−2.902 **	−1.545	−2.731 ***	−2.988 **	−2.909 **	**−2.564 ****	**−2.476 ****
PN-SMD 3	2.133 *	0.637	2.093 *	0.643	0.144	0.12	0.353	0.313
PN-SMD 4	1.578	/	1.844	−0.005	−0.446	−0.44	−0.204	−0.168
**PN-SMD 5 (good)**	−0.558	−2.694 **	/	−2.369 **	−2.327 **	−2.326 *	**−2.058 ***	**−1.973 ***
PN-SMD 6	2.634 **	1.42	3.227 **	1.564	0.724	0.697	0.938	0.943
PN-SMD 7	1.269	−0.288	1.353	/	−0.584	−0.574	−0.339	−0.313
PN-SMD 8	0.632	1.461	0.774	1.412	1.96	2.109*	1.714	1.565
**PN-SMD 9 (lonely)**	1.071	2.081 *	1.373	2.060 *	3.158 **	3.176 **	**2.577 ****	**2.347 ***
**PN-SMD 10 (discouraging)**	−2.407 *	−1.314	−2.053 *	−1.345	−1.444	−1.359	**−2.162 ***	**−2.100 ***
PN-SMD 11	−1.736	−0.435	−1.233	−0.498	/	−0.023	−0.645	−0.758
PN-SMD 12	−1.588	−0.46	−1.243	−0.513	−0.169	/	−0.897	−0.917
PN-SMD 13	−0.775	0.149	−0.588	0.113	0.637	0.876	/	−0.053
PN-SMD 14	−0.477	0.275	−0.269	0.249	0.794	0.933	0.264	/
Note. The detected DIF items were bold. * *p* < 0.05; ** *p* < 0.01; *** *p* < 0.001.								
